# The effect of hyperthermia with localised head and neck cooling on neuromuscular function

**DOI:** 10.1186/2046-7648-4-S1-A3

**Published:** 2015-09-14

**Authors:** Ralph Gordon, Neale A Tillin, Jamie Hall, Kelly-Anne Clifford, Christopher J Tyler

**Affiliations:** 1 Sports and Exercise Science Research Centre, Departemnt of Life Sciences, University of Roehampton, London, UK

## Introduction

Hyperthermia reduces volitional force production, voluntary muscle activation and agonist-electromyography (EMG) during a sustained maximal voluntary contraction (MVC) [1], [2]. This reduction in neuromuscular function may explain a reduced exercise capacity in the heat. Cooling of the neck has been shown to improve running capacity in the heat [3]; however the mechanism is unknown. The aim of the study was to investigate whether localised cooling of the head and neck during hyperthermia would affect neuromuscular function following 60 min of cycling in the heat.

## Methods

Fourteen male participants exercised on a cycle ergometer for 60 min at 50% VO_2max _in three experimental conditions; hot (35 °C, 50% rh; HOT), hot with head and neck cooling (35 °C, 50% rh; HOT_cooling_) and control (18 °C, 50% rh; CON). Immediately after the cycling bout, participants performed a 120-s sustained isometric MVC of the knee extensors of their dominant limb. Neuromuscular activation was assessed during the MVC at 5, 30, 90 and 120-s by superimposing supra-maximal triplet (3 impulses at 100 Hz) contractions by electrical stimulation of the femoral nerve, and calculating the central activation ratio (CAR). EMG amplitude (normalised to maximal M-wave) of the 3 superficial quadriceps heads was recorded throughout the MVC. Rectal temperature (T_re_) was measured throughout each condition.

## Results

**Figure 1:**
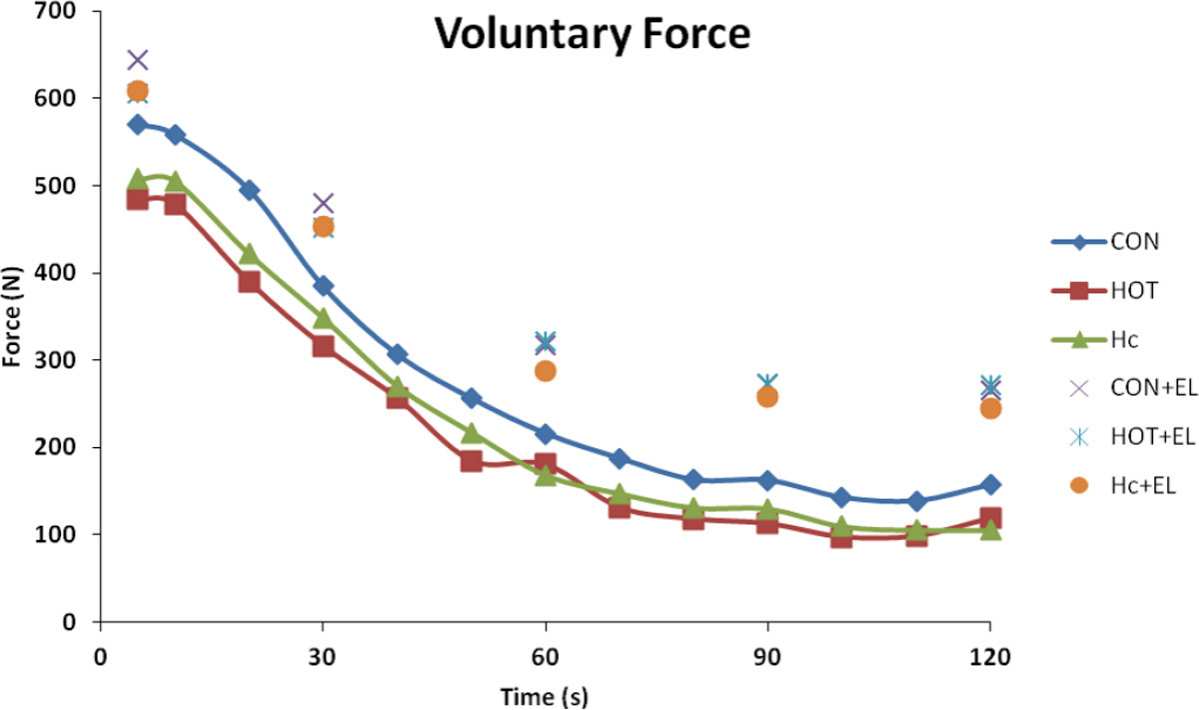
**Changes in force of the leg extensors during 120-s of isometric MVC in HOT, HOT_Cooling _CON**. Electrical stimulation (EL) was superimposed every 30s to assess central activation. Data are means for 14 subjects.

T_re _was raised in both the HOT (39.27 (0.52) °C) and HOT_cooling _(39.19 (±0.56) °C) trials vs CON (38.07 (0.28) °C) immediately post cycling (P <0.001) and remained elevated during the 120-s MVC. Force declined throughout the MVC in all conditions (Figure 1). The decline in force was on average 18 and 13.6% greater in HOT and HOT_cooling _respectively compared to CON (Figure 1; P <0.001 for both). This was similar for voluntary activation, with significant reductions in HOT vs CON trials across all time points (P <0.001). Normalised agonist EMG showed significant differences between HOT vs CON throughout the first 60-s of contraction; thereafter a reduced neural drive in all conditions was similar.

## Discussion

Cooling had no physiological effect on T_re _during hyperthermia trials. HOT appeared to show the greatest decline in voluntary force and was associated with a greater decline in CAR and normalised EMG in comparison to CON. The higher force output for HOT_cooling _may be explained by improved neural drive of the central nervous system to voluntarily activated muscles.

## Conclusion

Localised head and neck cooling improves neuromuscular function of the knee extensors during a sustained MVC under hyperthermic conditions.
